# Effects of Caloric Intake on Learning and Memory Function in Juvenile C57BL/6J Mice

**DOI:** 10.1155/2015/759803

**Published:** 2015-09-27

**Authors:** Bao-Lei Xu, Rong Wang, Li-Na Ma, Wen Dong, Zhi-Wei Zhao, Jing-Shuang Zhang, Yu-Lan Wang, Xu Zhang

**Affiliations:** ^1^Central Laboratory, Xuanwu Hospital, Capital Medical University, Key Laboratory for Neurodegenerative Disease of Ministry of Education, Beijing Geriatric Medical Research Center, No. 45 Changchun Street, Xicheng District, Beijing 100053, China; ^2^Department of Neurology, Beijing Anzhen Hospital, Capital Medical University, No. 2 Anzhen Road, Chaoyang District, Beijing 100029, China; ^3^Center of Alzheimer's Disease, Beijing Institute for Brain Disorders, Capital Medical University, Beijing 100069, China

## Abstract

Dietary composition may influence neuronal function as well as processes underlying synaptic plasticity. In this study, we aimed to determine the effect of high and low caloric diets on a mouse model of learning and memory and to explore mechanisms underlying this process. Mice were divided into three different dietary groups: normal control (*n* = 12), high-caloric (HC) diet (*n* = 12), and low-caloric (LC) diet (*n* = 12). After 6 months, mice were evaluated on the Morris water maze to assess spatial memory ability. We found that HC diet impaired learning and memory function relative to both control and LC diet. The levels of SIRT1 as well as its downstream effectors p53, p16, and peroxisome proliferator-activated receptor *γ* (PPAR*γ*) were decreased in brain tissues obtained from HC mice. LC upregulated SIRT1 but downregulated p53, p16, and PPAR*γ*. The expressions of PI3K and Akt were not altered after HC or LC diet treatment, but both LC and HC elevated the levels of phosphorylated-cAMP response element-binding protein (p-CREB) and IGF-1 in hippocampal CA1 region. Therefore, HC diet-induced dysfunction in learning and memory may be prevented by caloric restriction via regulation of the SIRT1-p53 or IGF-1 signaling pathways and phosphorylation of CREB.

## 1. Introduction

A variety of lifestyle factors contribute to cognitive decline in the elderly [1, 2]. In particular, dietary composition has an important role, with excessive caloric intake associated with accelerated aging of the brain. A high-fat, high-energy diet has been shown to impair learning and memory [3, 4], whereas caloric restriction may actually improve it [5]. Most of these studies, however, examined aged animals, and the effects of high or low calorie diets on brain function in juvenile animals remain poorly understood.

The mammalian nicotinamide-adenine dinucleotide-dependent deacetylase silent information regulator type 1 (SIRT1) plays a critical role in the regulation of normal cognitive function and synaptic plasticity and has been implicated in senescence processes [6]. In addition, SIRT1 expression has been linked to insulin sensitivity, whereas its downregulation is associated with insulin resistance [7]. Downstream effectors of the SIRT1 pathway include p53 [8], p16 [9], and peroxisome proliferator-activated receptor *γ* (PPAR*γ*) [10]. The phosphatidylinositol 3 kinase (PI3K)/Akt signal pathway has also been implicated in caloric restriction-mediated improvement of learning ability in mice [11]. Therefore, we hypothesized that dietary composition would alter learning and memory in juvenile mice, perhaps via the SIRT1 and/or PI3K/Akt signaling pathways.

## 2. Materials and Methods

### 2.1. Animals

In total, 36 6-week-old male and female C57BL/6J mice were used in this study. The mice were obtained from Laboratory Animal Center of the Academy of Military Medical Sciences, China, and weighed 16.1–25.1 g. All animal experiments were approved by the Animal Ethical Committee of Xuanwu Hospital.

### 2.2. Diets and Experimental Assignments

Prior to the start of the experiment, animals were adapted to the housing condition for 2 weeks Mice which were randomly divided into three groups: control (*n* = 12), high-caloric (HC) diet group (*n* = 12), and low-caloric (LC) diet group (*n* = 12). Animals in the control, HC, and LC groups were fed stock specific pathogen-free- (SPF-) level diet from the Laboratory Animal Center of the Academy of Military Medical Sciences, China, for 6 months. The composition of all diets was adjusted according to our preliminary experiments. The HC diet (30% caloric enhancement) was composed of 63% stock diet, 19% lard compound, 10% sucrose, and 8% isolated soy protein; LC diet (30% caloric reduction) was composed of 58% stock diet, 34% dietary fiber, and 8% isolated soy protein. All mice had free access to water. Animal body weight was measured every four weeks, and fasting blood-glucose level was examined once a month. Six months later, animals were sacrificed and cortex and hippocampus tissues were collected for Western blotting and real time PCR analysis.

### 2.3. Serological Test

Serum IGF-1 and serum insulin levels were quantitated using enzyme linked immunosorbent assay (ELISA) kits according to the manufacturer's instructions (Nanjing Jiancheng Bioengineering Institute, Nanjing, Jiangsu, China). Serum cholesterol and serum triglyceride levels were examined using a biochemical analyzer (AU400, Olympus, Center Valley, PA, USA).

### 2.4. Morris Water Maze Test

The Morris water maze task [12] was utilized to evaluate learning and memory function. The testing apparatus was provided by the Institute of Materia Medica, Chinese Academy of Medical Sciences (Beijing, China). Briefly, a circular pool of water (diameter: 120 cm, height: 30 cm) was maintained at 24°C + 1°C and contained four quadrants (designated northeast, northwest, southeast, and southwest). In the northeast quadrant, a platform was submerged under approximately 2.0 cm below the surface of the water. On the first day, mice were initially placed onto the platform for 10 s. Then, mice were placed in the water facing the pool wall in the southwest quadrant. The animals were allowed to swim for a maximum of 60 sec to find the hidden platform. If the animals failed to find the target, and they were led manually to the platform. The place navigation test was performed from day 2 to day 5. The time elapsed (escape latency) and the swimming distance were recorded. If the mouse failed to find the platform within the allotted time period, the mouse was given a score of 60 sec.

### 2.5. Real Time PCR

An RNA extraction kit was utilized to isolate RNA from brain tissue samples according to the manufacturer's instructions (Beijing ComWin Biotech Co., Ltd., Beijing, China). The HiFi-MMLV first-strand cDNA synthesis kit was used to reverse transcribe total RNA (Beijing ComWin Biotech Co., Ltd.), and an AB7500 sequence detection system was used to perform quantitative reverse transcriptase polymerase chain reaction (qRT-PCR) (Applied Biosystems, Carlsbad, CA, USA). PCR amplification was carried out using UltraSYBR Mixture (with Rox; Beijing ComWin Biotech Co., Ltd.) with the specific primers: SIRT1, forward, 5′-TATGACGCTGTGGCAGATTGTTATT-3′; reverse, 5′-CCACCGCAAGGCGAGCAT-3′; actin, forward, 5′-GCCTTCCTTCTTGGGTAT-3′; reverse, 5′-GGCATAGAGGTCTTTACGG-3′. Duplicate PCR reactions were performed at 95°C for 10 min and subjected to 45 cycles of 95°C for 15 s and 60°C for 60 s. For each condition, the experiment was replicated in triplicate. The relative expression from amplified RNA samples was calculated using the 2^−ΔΔCT^ method.

### 2.6. Western Blotting

Total protein from brain tissues was homogenized in lysis buffer containing 50 mM Tris-HCl (pH 7.4), 1 mM phenylmethanesulfonyl fluoride (PMSF), 0.1% leupeptin, and 0.5 M ethylenediaminetetraacetic acid (EDTA) (pH 8.0). Protein concentration was measured using a bicinchoninic acid (BCA) protein assay kit according to manufacturer's instructions (Beijing SiNoble Biotechnology Inc., Beijing, China). Equal amounts of protein were resolved by sodium dodecyl sulfate-polyacrylamide gel electrophoresis (SDS-PAGE) and transferred to a nitrocellulose membrane (Millipore, Billerica, MA, USA). Membranes were blocked in 5% w/v nonfat dry milk made in tris-buffered saline plus Tween-20 (TBS-T; 0.1% Tween-20) and incubated with the following primary antibodies at 4°C overnight: rabbit anti-PI3K (1 : 1000, Epitomics, Burlingame, CA, USA), rabbit anti-Akt (1 : 20,000, Cell Signaling, Beverley, MA, USA), rabbit anti-p53 (1 : 2000, Beijing TDY Biotech Co. Ltd.), rabbit anti-SIRT1 (1 : 2000, Abcam, Cambridge, UK), rabbit anti-PPAR*γ* (1 : 1000, Bioworld, St Louis Park, MN, USA), and mouse anti-GAPDH (1 : 20,000, Beijing TDY Biotech Co. Ltd.). After rinsing with TBS-T, membranes were incubated with either goat anti-rabbit or goat anti-mouse horseradish peroxidase- (HRP-) conjugated immunoglobulin (Ig)G (H + L) secondary antibodies (1 : 20,000 or 1 : 10,000, Beijing TDY Biotech Co. Ltd.) for 40 min at room temperature. Immunobands were visualized using enhanced chemiluminescence (ECL) kits according to the manufacturer's instructions (Merck Millipore, Darmstadt, Germany).

### 2.7. Immunohistochemical Analysis

Hippocampal tissue samples were fixed in 4% paraformaldehyde, dehydrated in an ethanol series (50%, 70%, 80%, 90%, 95%, and 100%), cleared in xylene, and embedded in paraffin. Paraffin-embedded tissues were coronally sectioned in an automatic tissue processor (Leica, Wetzlar, Germany) to yield 5 *μ*m thick sections. Sections were probed with rabbit primary antibody (1 : 1000) and goat anti-rabbit biotin-conjugated secondary antibody (Beijing Zhong Shan Golden Bridge Biological Technology Co. Ltd). The following primary antibodies were synthesized in the Bioactive Peptides Laboratory of Xuanwu Hospital (Beijing, China): anti-IGF-1, anti-PI3K, anti-phosphorylated-cAMP response element-binding protein (p-CREB), anti-SIRT1, anti-p16, anti-PPAR*γ*, and anti-p53 antibodies. Antibody binding was visualized with 3,3′-diaminobenzidine tetrahydrochloride (DAB) kit (Beijing Zhong Shan Golden Bridge Biological Technology Co. Ltd), and samples were counterstained with hematoxylin and eosin. Three slides were chosen at random from each animal for quantification. The number of immunopositive neurons in three visual fields per slide was quantitated with Image-Pro Plus software.

### 2.8. Statistical Analysis

Data were analyzed with SPSS17.0 software (Chicago, IL, USA). All data are presented as means ± standard deviation (SD) and analyzed using one-way analysis of variance (ANOVA). Repeated measures two-way ANOVA was used to compare escape latency and swimming distances among the three groups. *P* < 0.05 or *P* < 0.01 was considered significantly different.

## 3. Results

### 3.1. Effects of Caloric Intake on Physiological Indicators

**Figure 1:**
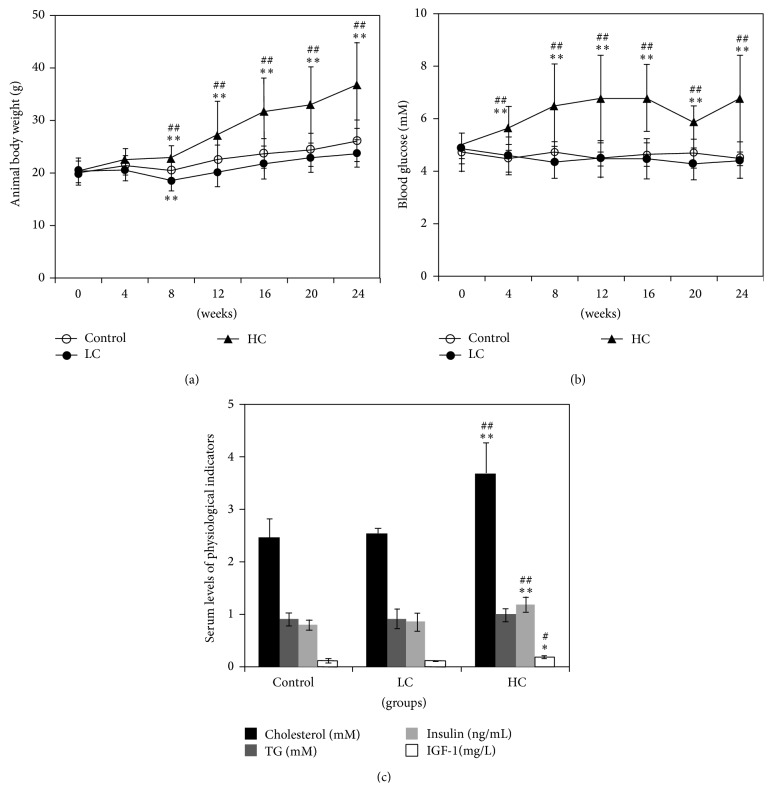
Physiological changes in mice fed different diets. Mice were fed normal, HC, or LC diet for 6 months. Animal body weight (a); blood glucose level (b); and serum cholesterol, serum triglyceride (TG), serum insulin, and serum IGF-1 levels (c) are shown. ^*^ *P* < 0.05, ^**^ *P* < 0.01 compared with control; ^##^ *P* < 0.01 compared with LC. *N* = 6 for each group.

First, we examined the physiological differences in the groups fed normal, HC, and LC diets. As shown in Figure 1(a), mice in the HC group demonstrated time-dependent elevation in body weight that was significantly greater than the other two groups by 8 weeks (*P* < 0.01). Body weight in the LC group was attenuated relative to control at 8 weeks (repeated measured two-way ANOVA, *P* < 0.05). At 4 weeks, blood glucose level and serum levels of cholesterol, insulin, and IGF-1 were significantly elevated in the HC diet (*P* < 0.01 compared with the other two groups) (Figure 1(b)). There were no detectable changes in serum triglyceride levels among the three groups (*P* > 0.05) (Figure 1(c)). Mice in the LC group did not exhibit significant alterations in blood glucose or serum levels of cholesterol, insulin, or IGF-1 relative to control (*P* > 0.05) (Figures 1(b) and 1(c)).

### 3.2. Effects of Caloric Intake on Learning and Memory Abilities in Mice

**Figure 2:**
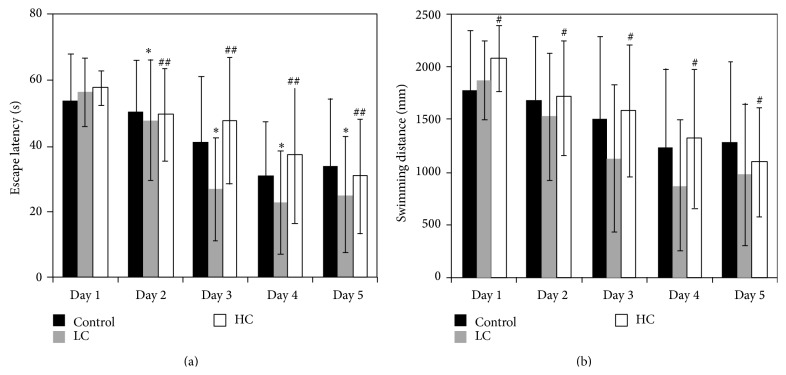
Morris water maze performance in mice fed different diets. Escape latency (a) and swimming distance (b) of mice in normal control, HC, or LC groups are shown. ^*^ *P* < 0.05 compared with control; ^#^ *P* < 0.05, ^##^ *P* < 0.01 compared with LC. *N* = 12 for each group.

The Morris water maze is a well-established model of spatial learning and memory. Mice in the control and two experimental groups were trained on this task in order to assess the potential differential effects of high and low caloric intake on regulation of learning and memory processes. In all groups, escape latency and swimming distance gradually decreased with repeated trials over five days, and there was a significant difference among the three groups (*F* = 4.376; *P* < 0.05). Animals in the LC group exhibited a significantly shorter escape latency than control (*P* < 0.05) (Figure 2(a)), whereas animals in the HC group demonstrated significantly increased escape latency and swimming distance relative to the LC group (*P* < 0.01 or *P* < 0.05) (Figures 2(a) and 2(b)). These results suggested that low caloric intake may increase learning and memory function, whereas high caloric intake may impair learning and memory.

### 3.3. SIRT1 Signaling Pathway in Mice Fed Different Caloric Diets

**Figure 3:**
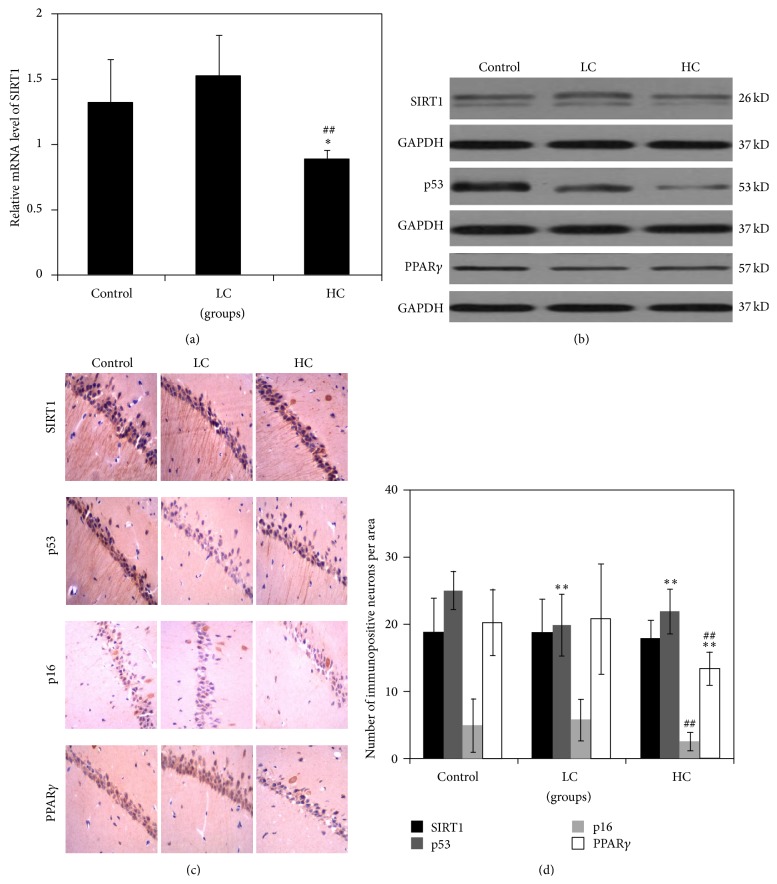
Expression of SIRT1 pathway regulators. The mRNA expression of SIRT1 in hippocampal and cortical tissues of mice examined by RT-PCR (a). The protein expressions of SIRT1, p53, p16, and PPAR*γ* assessed with Western blot (b) and immunohistochemistry (c), respectively. Magnification: ×400. The number of immunopositive neurons per area in hippocampal CA1 region was counted (d). ^**^ *P* < 0.01 compared with control; ^##^ *P* < 0.01 compared with LC. *N* = 6 for each group.

In order to further understand the molecular mechanisms underlying alterations in learning and memory in mice fed different caloric diets, we investigated the SIRT1 signaling pathway. As shown in Figures 3(a) and 3(b), the levels of SIRT1 mRNA and protein were significantly downregulated in brain tissue from HC mice relative to the other two groups (mRNA, control: 1.31 ± 0.34; LC: 1.52 ± 0.32; HC: 0.88 ± 0.08; *P* < 0.05). The expressions of downstream targets of the SIRT1, including p53, p16, and PPAR*γ*, were decreased in the HC group as well (Figure 3(b)). Similar findings were observed in immunohistochemical analyses of these targets (Figures 3(c) and 3(d)). Although animals in the LC group exhibited a slight upregulation in the level of SIRT1 mRNA and protein, the difference was not statistically different (Figures 3(a) and 3(b)). The number of p53-positive neurons, however, was reduced in the hippocampus of LC mice relative to control (*P* < 0.01) (Figures 3(c) and 3(d)). These data suggest that HC may affect learning and memory by inhibiting SIRT1, whereas LC may be neuroprotective by positively regulating the SIRT1-p53 pathway.

### 3.4. IGF-1-PI3K/Akt Signaling Pathway in Mice Fed Different Caloric Diets

**Figure 4:**
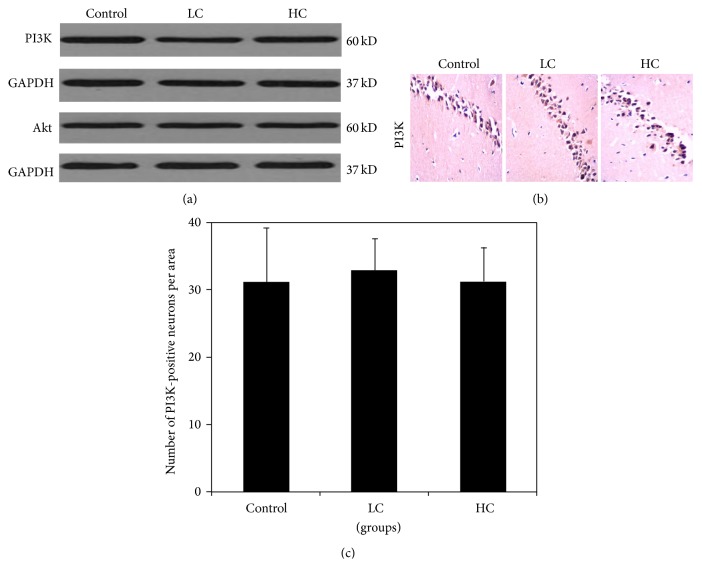
Expressions of IGF-1-PI3K/Akt pathway regulators. Protein expressions of PI3K and Akt assessed using Western blot (a). Immunohistochemical analysis of PI3K, p-CREB, and IGF-1 expression in hippocampal CA1 region (b and c). Magnification: ×400. The number of immunopositive neurons per area in hippocampal CA1 region was counted. ^**^ *P* < 0.01 compared with control. *N* = 6 for each group.

Since PI3K/Akt signaling pathway has been implicated in insulin-mediated regulation of learning and memory function, we also investigated the expression of IGF-1, PI3K, Akt, and p-CREB in brain tissue of mice fed different diets. There was no significant difference in the protein expression of PI3K or Akt (Figure 4(a)). In addition, the number of PI3K-positive neurons in CA1 of hippocampus was not altered in the HC or LC groups relative to control (*P* > 0.05) (Figures 4(b) and 4(c)). The expression of p-CREB was significantly upregulated with LC treatment (*P* < 0.01) (Figures 4(b) and 4(c)). Additionally, both LC and HC elevated the number of IGF-1-positive neurons in hippocampal CA1 region (*P* < 0.01) (Figures 4(b) and 4(c)). Although the activities of IGF-1 and p-CREB were altered by diet, our findings suggest that the PI3K/Akt signal pathway is unlikely to be involved in high or low caloric diet-mediated changes in learning and memory.

## 4. Discussion

A diet high in fat during midlife has been linked to cognitive impairment later in life [13]. Although many studies have addressed this phenomenon in aged animals, little evidence is available in young animals. Here, we investigated the effects of high-caloric and low-caloric diets on learning and memory abilities in juvenile (6-week-old) mice and explored the underlying signaling pathways involved.

We found that a six-month long HC diet greatly increased body weight, blood glucose, serum cholesterol, serum insulin, and serum IGF-1 and impaired learning and memory function in mice. Although LC diet did reduce body weight, the levels of other physiological indicators were not altered. The LC diet did prevent impairments in learning and memory dysfunction. Consistent with these findings, it was shown that short-term administration of a high-fat diet (55% kCal from fat) impaired cognitive function in rats [3]. In contrast, caloric restriction has been associated with enhanced memory consolidation and facilitation of synaptic plasticity [14]. Taken together, these observations suggest that long-term HC diet likely impairs learning and memory function, even in juvenile animals, and that LC diet, while not altering levels of physiological indicators, can improve learning and memory.

SIRT1 is known to regulate processes underlying longevity, and its role in delaying senescence has been extensively investigated [15]. Previously, it was shown that caloric restriction upregulated SIRT1 expression, and this may be an essential event of lifespan elongation under caloric restriction [16]. Indeed, we demonstrated that SIRT1 as well as several of its substrates, including p53, p16, and PPAR*γ*, is downregulated in HC diet fed mice. In LC diet mice, SIRT1 expression was upregulated, but p53 expression was downregulated. It is possible that other downstream factors of SIRT1 may participate in LC-mediated SIRT1 activation. Future studies will continue to investigate the molecular mechanisms involved in the regulation of the SIRT1 signaling pathway in HC or LC diet fed mice.

Furthermore, we investigated the potential involvement of IGF-1-PI3K/Akt signal pathway in diet induced changes in learning and memory. There were no significant differences in the expression of PI3K or Akt among the three groups. However, we did not measure the levels of phosphorylated Akt in this current study. Therefore, we could not exclude the possibility that Akt activation might be involved in this process. Indeed, previous study indicated that Sirt1 had the ability to activate Akt* via* Akt deacetylation during tumorigenesis [17]. In our future study, we will continue to explore the potential involvement of Akt activation in this process. Interestingly, both IGF-1 and p-CREB levels were upregulated in the CA1 region of hippocampus of mice fed either HC or LC diet. It is possible that this increase in IGF-1 and p-CREB serves as a compensatory mechanism in the HC group, while LC might prevent learning and memory deficits by increasing the activity of IGF-1 and p-CREB. Consistent with this, the level of p-CREB was higher in LC mice relative to HC mice. We did not, however, rule out the possible participation of other factors or regulators in the IGF-1-PI3K/Akt signal pathway in the above process. Future studies will continue to explore the molecules involved in LC-mediated neuroprotection and HC-mediated brain damage.

## 5. Conclusions

In the present study, we found that long-term HC diet impaired learning and memory function, possibly via blockade of the SIRT1 signaling pathway. In addition, long-term LC diet may improve learning and memory by upregulating the expression of SIRT1, IGF-1, and p-CREB. Our study provides important details regarding alterations in brain function and chemistry following chronic changes in dietary intake and suggests that SIRT1, IGF-1, or p-CREB and LC diet may be promising therapeutic targets for the prevention of learning and memory impairments in the young and elderly.
